# The safety, tolerability, and pharmacokinetic profile of GSK2838232, a novel 2nd generation HIV maturation inhibitor, as assessed in healthy subjects

**DOI:** 10.1002/prp2.408

**Published:** 2018-06-05

**Authors:** Mark Johnson, Roxanne C. Jewell, Amanda Peppercorn, Elizabeth Gould, Jianfeng Xu, Yu Lou, Matthew Davies, Sandra Baldwin, Allan R. Tenorio, Matthew Burke, Jerry Jeffrey, Brian A. Johns

**Affiliations:** ^1^ GlaxoSmithKline Research Triangle Park NC USA; ^2^ GlaxoSmithKline Cambridge MA USA; ^3^ GlaxoSmithKline Collegeville PA USA; ^4^ Parexel Research Triangle Park NC USA; ^5^ GlaxoSmithKline Stockley Park NC USA; ^6^ GlaxoSmithKline Ware UK; ^7^ GlaxoSmithKline King of Prussia PA USA

**Keywords:** GSK2838232, healthy Subjects, HIV maturation inhibitor, pharmacokinetics, safety

## Abstract

This work aimed to assess the safety, tolerability, pharmacokinetics (PK), and relative bioavailability of GSK2838232, an investigational HIV maturation inhibitor. GSK2838232 was administered over four dose‐escalation studies in healthy subjects which assessed single oral doses (5‐250 mg) and repeat doses (up to 200 mg once or twice daily) ±100 mg ritonavir (RTV) once daily. GSK2838232 administration (up to 250 mg) to 124 subjects across four studies resulted in few mild adverse events (AEs) with similar frequencies to placebo. There were no clearly identified drug‐related AEs. GSK2838232 tested fasted was quickly absorbed with a *t*_max_ of 2‐3 hours. With food, the absorption was delayed and more variable, with ~60% increase in AUC and *C*_max_. Overall, following single doses GSK2838232 AUC and *C*_max_ generally exhibited proportional PK from 50 to 100 mg dose without RTV and from 50 to 250 mg with RTV and following repeated doses of 20‐200 mg with RTV. In relative bioavailability studies, a micronized formulation was found to be suitable for development. At steady state, RTV increased GSK2838232 AUC and *C*_max_ by 10‐ and 3‐fold, respectively. Half‐life was prolonged from ~17 hours nonboosted to ~34 hours with RTV. This boosting effect was also seen in repeat‐dose GSK2838232 studies, which achieved the targeted plasma exposure with GSK2838232 as a once‐daily regimen of up to 200 mg with RTV. The results of these studies demonstrated a favorable safety and PK profile for GSK2838232 and support its investigation for the treatment of HIV infection.

**Abbreviations**
AEs: adverse events
API: Active Pharmaceutical Ingredient
LLQ: lower limit of quantification
PiB: powder‐in‐bottle
PK: pharmacokinetics
SAE: serious adverse event
SDD: Spray Dried Dispersion

## 1 INTRODUCTION

Combination antiviral therapy with inhibitors of human immunodeficiency virus type 1 (HIV‐1) protease, integrase, entry, and reverse transcriptase has decreased HIV‐related morbidity and mortality over the last 20 years. Emerging multi‐class drug‐resistant viral strains and long‐term toxicities with current antiretrovirals warrant continued development of new classes of drugs targeting different stages of the HIV‐1 viral life cycle.^1^ The inhibition of the maturation process of HIV‐1 is a novel point of intervention for drug development, distinct from viral entry and the protease, reverse transcriptase, or integrase enzyme inhibitors.^2^

GSK2838232 is a maturation inhibitor being developed for the treatment of human HIV‐1. The HIV‐1 gag open reading frame (ORF) encodes for the majority of structural proteins required for virion assembly and is expressed as a polyprotein in an infected‐ and virus‐producing cell. Once the immature virion buds from the producer cell, HIV protease cleaves the gag polyprotein into the individual structural proteins. These cleavage events occur in a highly ordered fashion, culminating with a final cleavage of p25, termed maturation, that results in p24, the capsid protein that gives HIV the familiar bullet‐shaped virion core morphology. Various betulin triterpene‐based small molecules have shown antiviral activity by preventing the p25 to p24 cleavage step. Bevirimat (BVM) was the first maturation inhibitor to demonstrate antiviral activity in the clinic, but it was terminated due to a variety of developmental challenges.^3^ Key limitations of BVM were a lack of efficacy against naturally occurring polymorphisms in the HIV gag region, specifically the capsid (CA) & Sp1 regions, and a significant decrease in potency in the presence of human serum. When compared to BVM, another maturation inhibitor, BMS‐955176 (later known as GSK3532795), demonstrated greater and dose‐related anti‐HIV potency, with no obvious safety concerns in Phase IIa clinical studies.^4^ Unfortunately, this drug was terminated in Phase IIb clinical development because of concerns over safety/GI tolerability and viral resistance.^5^

**Figure 1:**
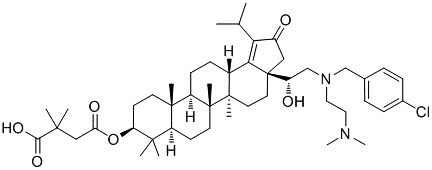
Structure of GSK2838232 (MW 809.6)

GSK2838232 (Figure 1) is derived from the triterpene betulin natural product that has demonstrated an excellent virological profile against a panel of viruses and retains activity to viruses with key resistance mutations.^6^ The IC90 in cell‐based assays has been calculated as 6.4 nmol/L (5 ng/mL), and there was no significant shift in this value in assays to which human serum albumin was added, even though plasma protein binding was assessed in vitro as >99.9% across all species. This value (termed PA‐IC90) was considered a minimal threshold to achieve in Phase I studies, prior to evaluation in HIV‐infected subjects.

In preclinical studies, GSK28382232 was shown to have low to moderate oral bioavailability (6%‐40%, depending on species and formulation) and was excreted via bile (primarily as metabolites) or in feces (primarily unabsorbed drug). The major compound in plasma circulation was parent compound. GSK2838232 is subjected to metabolism via multiple pathways, including Phase I oxidation (primarily by cytochrome P450 [CYP] 3A4) and Phase II glucuronidation, with negligible urinary excretion of drug‐related material (≤1% administered dose). Metabolite profiles observed preclinically were qualitatively similar to those observed in human plasma.

The current report presents the results from four Phase I dose escalation studies in healthy subjects to ascertain the safety, tolerability, and single‐ and repeat‐dose pharmacokinetics (PK) of GSK2838232 with and without ritonavir (RTV) as well as the relative bioavailability of suspension and solid oral dosage formulations and the impact of food.

## 2 MATERIALS AND METHODS

Each one of the four studies described were conducted and completed at a single center in the United States (Parexel International, Baltimore, MD) per the ethical principles of “good clinical practice” (GCP) and the Declaration of Helsinki after obtaining a written informed consent from each subject. The protocols and amendments were approved by Aspire Investigational Review Board (Santee, California, USA).

### 2.1 Study population and design

For all four studies, the inclusion/exclusion criteria allowed for the enrollment of healthy men and women of nonchild bearing potential aged between 18 and 55 years of age with a body weight ≥ 50 kg for men and ≥ 45 kg for women, body mass index (BMI) within the range 18.5 to 31.0 kg/m^2^ (inclusive), and creatinine clearance >80 mL/min at time of screening. Other screening assessments included 24‐hour Holter monitoring and baseline echocardiograms.

### 2.2 Study designs

#### 2.2.1 Single‐ascending‐dose escalation study (5‐100 mg) and food effect—spray dried dispersion formulation (Study HMI116787, NCT01802918)

This study was a first‐in‐human, double‐blind, randomized, placebo‐controlled, single dose escalation study of GSK2838232 in healthy subjects conducted in two sequential cohorts with four dosing visits in each cohort. At each visit in each cohort, 6 out of the 8 subjects were randomized to receive the active dose, and 2 out of the 8 subjects were randomized to placebo. Cohort 1 tested escalating doses of 5, 10, and 20 mg GSK2838232 as a Spray Dried Dispersion (SDD) delivered as a powder‐in‐bottle (PiB) suspension; Cohort 2 tested the same formulation at doses of 50 and 100 mg, the effect of a high‐fat breakfast on a 50 mg dose, and the impact of steady‐state dosing of 100 mg once‐daily (QD) RTV on the PK of a single 10 mg dose of GSK2838232 (administered on Day 10 of the 12 days of RTV dosing). All doses were administered in a fasted state, except in the food effect portion of the study.

#### 2.2.2 Single‐ascending‐dose escalation study (100‐200 mg)— spray dried dispersion and active pharmaceutical ingredient formulations (study 200912, NCT02289482)

This was a double‐blind, randomized, placebo‐controlled, single‐dose escalation study to investigate the safety, tolerability, and PK of higher single doses of GSK2838232 (Part A) and to evaluate the relative bioavailability of the SDD and crystalline Active Pharmaceutical Ingredient (API) of GSK2838232 (Part B) in healthy subjects dosed as PiB.

Part A was originally planned as a double‐blind, randomized, placebo‐controlled, 4‐period, single‐dose escalation design. Subjects were randomized 3:1 to receive GSK2838232 or placebo. Periods 1 and 2 investigated dosing with 200 mg SDD or nonmicronized API. Further planned dosing of RTV‐boosted single doses of 20 mg and 50 mg SDD was not performed due to early termination of the study to evaluate a serious adverse event (SAE) of a cardiovascular nature (see Safety discussion).

Part B was conducted in a separate group of subjects and was not dependent on the results of Part A. This was a randomized, open‐label, 3‐period, crossover design to assess the relative bioavailability of the nonmicronized API PiB versus the original SDD formulation at a 100 mg dose level (Periods 1 and 2). Based on an interim analysis of these data confirming that crystalline API was viable, a single dose of 20 mg API was assessed after RTV predosing for 10 days. Subjects were randomized 1:1 to each dosing sequence.

#### 2.2.3 Multiple‐ascending‐dose escalation study (20‐50 mg) ± rtv ‐ spray dried dispersion formulation (study 200207, NCT02289495)

This study was conducted in parallel with Study 200912 and was planned as a two‐part, double‐blind, placebo‐controlled, repeat dose escalation study of GSK2838232 with and without RTV for 8‐11 days. Subjects were randomized 3:1 to receive GSK2838232 or placebo. Part A was designed to evaluate GSK2838232 given alone for 8 days (20 mg and 50 mg GSK2838232 once‐daily (QD) for 8 days, respectively) and Part B was designed to evaluate GSK2838232 when co‐administered with 100 mg RTV QD for 11 days. Because of the occurrence of an SAE that was eventually determined to be unlikely to be related to GSK2838232 (see Safety discussion), the study was terminated early after one cohort in Part B (10 mg GSK2838232 + 100 mg RTV QD) had completed only 5 days of the planned 11‐day regimen.

#### 2.2.4 Single‐, multiple‐dose escalation, and relative bioavailability (50‐250 mg) ± RTV‐powder‐in bottle API versus powder blend capsule formulation (Study 204953, NCT02795754)

This study was planned as an extension of the previously conducted single‐ and multiple‐dose escalation studies described above. It was a double‐blind, placebo‐controlled, single and repeat dose escalation study of micronized API GSK2838232 with RTV conducted in three parts (Part 1A, Part 1B, and Part 2).

In Part 1A, a cohort of subjects was given single doses of 50, 100, and 250 mg of GSK2838232 with RTV, following 2 days of RTV predosing, in a sequential dose‐escalation design. Part 1B studied the relative bioavailability of GSK2838232 given as 100 mg (2 × 50 mg) capsules versus the reference API (PiB) in a two‐period crossover design with RTV, following 2 days of RTV predosing. In Period 3, subjects took the capsule formulation and RTV with a moderate fat meal (30% of calories from fat).

In Part 2, GSK2838232 was given to separate cohorts as once‐daily 20, 50, 100, and 200 mg doses with RTV for 11 days, with GSK2838232 and RTV dosing starting at the same time (i.e., no RTV predosing). GSK2838232 was administered as PiB for the 20 and 50 mg cohorts and as the capsule formulation for the 100 and 200 mg cohorts. Another separate cohort of subjects received GSK2838232 alone (i.e., unboosted) at a dose of 200 mg twice‐daily (BID; given every 12 hours) for 11 days using the capsule formulation to assess trough concentrations without RTV co‐administration.

### 2.3 Bioanalysis

Blood samples were taken via an indwelling cannula or by direct venipuncture, collected into a di‐potassium ethylenediaminetetraacetic acid (K_2_ EDTA) tube, and placed on wet ice. Samples were centrifuged at approximately 1500*g* for 10 minutes at approximately 4°C. Exactly 500 μL of the resulting plasma was transferred into labeled 1.4 mL Matrix TrakMate Tubes containing exactly 500 μL of 50 mmol/L citrate buffer, pH 4.0, and mixed well. When 500 μL of plasma was not able to be transferred, the ratio of plasma to citrate buffer was kept at 1:1. Buffered plasma samples were kept on ice and frozen at −70°C within 1 hour of collection. Plasma samples were analyzed for GSK2838232 using a validated analytical method based on protein precipitation, followed by high performance liquid chromatography with tandem mass spectrometry (HPLC/MS/MS) analysis.

Extracts were analyzed using HPLC (Shimadzu, Nexera, 30 series) and tandem mass spectrometry detection (HPLC‐MS/MS). The mass spectrometer used was an API‐5500 using IonSpray interface (500 V) in positive mode monitoring. The mass transitions were 809.5 to 255.1 and 816.5 to 262.1 for GSK2838232 and [2H7] GSK2838232 (the isotopically labeled internal standard), respectively. The chromatographic conditions utilized a Waters Acquity UPLC BEH C18 column (50 × 2.1 mm, 1.7 μm) with mobile phases consisting of 1 mmol/L ammonium formate:formic acid (100:0.005) (mobile phase A) and acetonitrile:formic acid (100:0.1) (mobile phase B) at a flow rate of 0.6 mL/min, with pump B at 63% held for 2 minutes. The assay was validated to have precision (%RSD) of 0.7%‐6.5% (within‐run) and 1.0%‐5.9% RSD (between‐run) and with accuracy (%bias) of 0.0%‐1.6%. The presence of ritonavir did not affect the quantification of GSK2838232 in acidified human plasma. The lower limit of quantification (LLQ) was 0.5 ng/mL, using a 50 μL aliquot of K_2_EDTA acidified plasma. The higher limit of quantification (HLQ) was 500 ng/mL.

Urine was not collected in any of the clinical studies, as preclinical evaluation indicated negligible renal excretion (≤1% of administered dose) of parent or drug‐related material.

### 2.4 Pharmacokinetic data analysis

For all four studies, the primary objectives were to describe the safety, tolerability, and pharmacokinetics of single and repeated doses of GSK2838232 with or without co‐administration of RTV. No formal statistical hypotheses were tested. All data were descriptively summarized.

The PK parameters were calculated by standard noncompartmental analysis based on actual sampling times using WinNonlin Version 6.3 or higher. Estimation of AUC used the linear up/log down trapezoidal rule.

For the assessment of relative bioavailability of the capsule formulation compared to the PiB suspension and of the food effect using the capsule formulation (all with RTV), PK parameters were separately analyzed for each treatment using a mixed effects model with fixed effect terms for period and treatment following log_e_ transformation. Subject was treated as a random effect in the model. Point estimates and their associated 90% CIs were constructed for the differences in PK parameter values between the test and reference treatments. The point estimates and their associated 90% CIs were then back‐transformed to provide point estimates and 90% CIs for the ratios of PK parameters from test and reference treatments on the original scale.

## 3 RESULTS

### 3.1 Subject disposition and demographics

Across the four studies, a total of 124 healthy subjects were exposed to GSK2838232 or placebo, of whom 9 subjects were female. The subject demographics were similar across all 4 studies with respect to age, weight and height (Table 1). Of the 124 subjects, one was withdrawn in Study 200207 (NCT02289495) due to an SAE of a cardiovascular nature that was eventually determined to be unlikely to be related to GSK2838232 (detailed in safety results section), and two subjects were withdrawn due to adverse events (AEs) in Study 204953 (NCT02795754), neither of which was considered related to GSK2838232.

**Table 1:** Demographics (Mean ± SD)

Study (N)	Age (years)	Height (cm)	Weight (kg)
HMI116787 (N = 17)	31.4 ± 8.40	177.4 ±8.82	78.06 ± 12.02
200912 (N = 20)	39.5 ± 9.56	176.6 ± 6.24	82.48 ± 10.69
200207 (N = 24)	41.0 ± 7.81	178.8 ± 5.67	85.69 ± 7.66
204953 (N = 63)	39.4 ± 8.85	175.1 ± 7.30	79.21 ± 9.14

### 3.2 Pharmacokinetic results

#### 3.2.1 Single‐dose pharmacokinetics ±RTV

**Figure 2:**
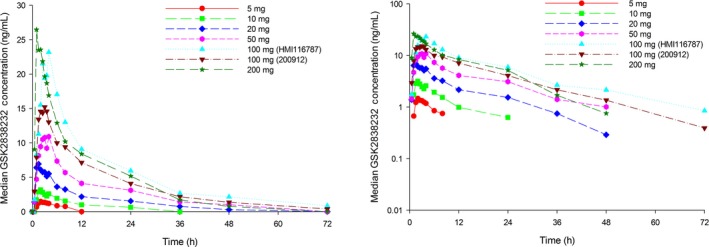
Median plasma concentration‐time profiles after single‐doses of GSK2838232 (SDD ^1^, Powder in Bottle)^2^. ^1^Spray Dried Dispersion (SDD) formulation; ^2^All data from HMI116787 unless stated

After single dose administration of GSK2838232 (fasted) as the SDD formulation, concentration‐time plots appeared to show one‐compartment pharmacokinetics, and the area under the concentration curve (AUC) and maximal plasma concentrations (*C*_max_) were proportional to dose over the dose range of 5‐100 mg (Figure 2, Table 2), with a less‐than‐proportional increase when the dose was increased to 200 mg. Terminal phase half‐life (t½) values were approximately 17‐19 hours at GSK2838232 doses of 50 mg or higher when administered without RTV.

**Table 2:** Single dose pharmacokinetics of GSK2838232 (Fasted) with and without ritonavir

Dose (Study)	N	*C*_max_ (ng/mL)	*t*_max_ (h)	AUC(0‐∞) (ng.h/mL)	t½ (h)	C24 (ng/mL)	CL/F (L/h)
Without ritonavir							
5 mg (HMI116787)	8	1.52 (1.02, 2.27)	2.24 (1.50‐8.00)	ND	ND	0 (0‐0.60)	ND
10 mg (HMI116787)	8	3.49 (2.98, 4.09)	1.76 (1.50‐4.00)	ND	ND	0.63 (0‐0.86)	ND
20 mg (HMI116787)	8	8.09 (6.28, 10.4)	1.75 (1.00‐4.02)	104 (78.8, 136)	14.1 (10.5, 18.9)	1.54 (0.92‐2.91)	193 (147, 254)
50 mg (HMI116787)	6	13.1 (8.14, 21.1)	2.02 (1.50‐4.00)	195 (154, 246)	17.4 (13.3, 22.8)	3.11 (2.03‐4.49)	256 (203, 324)
100 mg (HMI116787)	6	23.7 (15.7, 35.6)	2.75 (1.50‐4.00)	385 (299, 496)	18.8 (15.6, 22.6)	5.93 (3.92‐9.81)	260 (202, 335)
100 mg (200912, SDD)	12	18.7 (12.0, 29.2)	2.75 (1.00‐4.00)	265 (179, 391)	16.9 (13.6, 20.9)	3.87 (1.30‐10.6)	378 (256, 559)
100 mg (200912, API)	12	6.15 (4.24, 8.93)	3.64 (1.50‐6.00)	91.7 (56.4, 149)	12.5 (9.12, 17.1)	2.02 (0.59‐3.84)	1090 (670, 1774)
200 mg (200912, SDD)	6	29.3 (26.3, 32.7)	1.75 (1.00‐3.50)	381 (290, 501)	16.6 (12.7‐21.6)	5.18 (3.84‐9.16)	525 (399, 690)
200 mg (200912, API)	6	13.6 (8.77, 21.2)	2.76 (2.00‐6.02)	189 (122, 292)	15.9 (11.1, 22.7)	2.85 (1.19‐3.91)	1060 (686, 1638)
With Ritonavir							
10 mg (HMI116787)	6	9.10 (6.85, 12.1)	4.00 (2.48‐6.00)	389 (285, 531)	33.7 (27.3, 41.5)	4.64 (3.48‐8.64)	25.7 (18.8, 35.1)
20 mg (200912, API)	12	4.89 (3.34,7.16)	5.01 (2.00‐8.00)	224 (159,316)	41.1 (35.1,48.3)	2.56 (0.89‐5.64)	89.2 (63.3, 126)
50 mg (204953)	8	15.7 (10.8, 23.0)	3.00 (2.50‐6.02)	444^a^ (312, 632)	22.1^1^ (18.6, 26.3)	7.26 (3.18‐9.61)	113^a^ (79.1, 160)
100 mg (204953)	6	24.7 (19.8, 30.9)	5.00 (2.50‐12.00)	846 (668, 1072)	17.9 (15.5, 20.6)	13.2 (8.64‐17.4)	118 (93.3, 150)
250 mg (204953)	5	63.0 (41.7, 95.2)	5.98 (2.00‐12.05)	1686 (1126, 2525)	17.9 (16.0, 20.0)	24.0 (16.0‐32.0)	148 (90.0, 222)

All data are presented as geometric mean (95% confidence intervals) except for *t*_max_ and C24, presented as median (range).

ND, not determined (Note: AUC(0‐t) was used for ±RTV comparison in HMI116787). Formulations used: SDD PiB in Studies HMI116787 and 200912; API PiB in Studies 200912 and 204953.

a n = 7.

**Figure 3:**
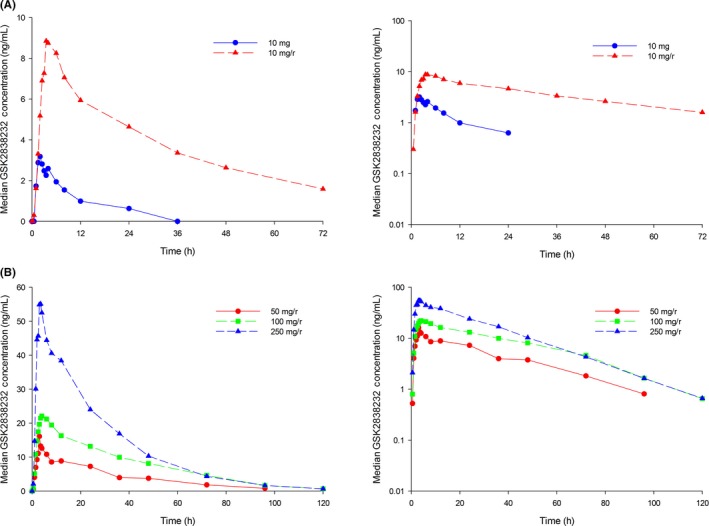
Median Plasma Single Dose Concentration‐Time Profiles of GSK2838232 with and without Ritonavir (A) 10 mg ± Ritonavir (HMI116787)^1^(B). 50‐250 mg with Ritonavir (204953)^2 1^Ritonavir was administered for 12 days, 10 mg GSK2838232 was administered on Day 10 2. Ritonavir was administered for 2 days prior to GSK2838232 dosing

Administration of RTV for 10 days prior to dosing with 10 mg GSK283832 SDD (Study 116787) resulted in an approximately 10‐fold increase in AUC(0‐t) and 3‐fold and 6.4‐fold increases in *C*_max_ and 24‐h concentrations (C24), respectively (Table 2, Figure 3A). In addition, *t*½ was approximately doubled, consistent with the reduced metabolic clearance. This finding was duplicated with a 20 mg nonmicronized API dose (Study 200912), where repeated doses of RTV to steady state had a significant impact on single dose GSK2838232 AUC, *C*_max_, and *t*½ values (Table 2).

In Study 204953, observed exposures in the presence of RTV appeared to be generally dose proportional in GSK283832 up to 250 mg (Figure 3B).

### 3.3 Multiple‐dose pharmacokinetics

The PK profile of unboosted GSK2838232 after multiple dosing for 8 days was as expected based on single dose data. AUC and *C*_max_ appeared slightly less than dose‐proportional between 20 mg and 50 mg GSK2838232 given QD (approximately a 2fold increase after a 2.5‐fold dose). The absorption profile was also similar to prior studies, with *C*_max_ occurring approximately 2‐3 hours after dosing. The accumulation ratios following repeated daily dosing with 50 mg GSK2838232 based on AUC, *C*_max_, and Cτ were 1.2, 1.1, and 1.4, respectively. The time to steady state was 3 days when administered unboosted. Variability in the PK parameters was 30%‐40% CV. The *t*½ value of GSK2838232 without boosting was approximately 15 hours.

**Figure 4:**
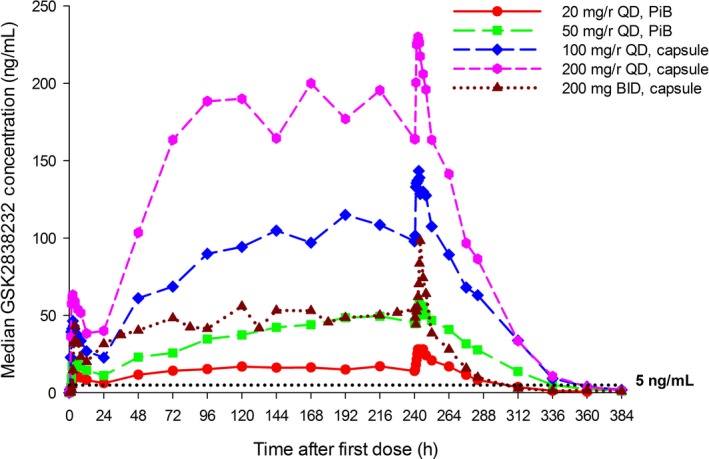
GSK2838232 Median Plasma Concentration‐Time Profiles with and without Ritonavir after Multiple Doses through Day 11—Study 204953. PA‐IC90 = protein‐adjusted 90% inhibitory concentration (5 ng/mL)

In Study 204953, a cohort of subjects was given 200 mg GSK2838232 BID without RTV. These results indicated that the overall steady‐state C24 levels of the 200 mg BID dose without RTV was similar to the 50 mg QD dose of GSK2838232 with RTV (Figure 4).

Multiple dosing of the micronized API form of GSK2838232 QD for 11 days in the presence of RTV appeared to be dose proportional through 200 mg with either powder‐in‐bottle or given as capsules (Study 204953, Table 3, Figure 3). The accumulation ratio in the presence of RTV was between 2‐ and 4‐fold, consistent with the reduction in CYP3A4‐mediated metabolic clearance of GSK2838232. The time to steady state was 4‐7 days when given with RTV. The *t*½ value of GSK2838232 in the RTV‐boosted cohorts was 18‐22 h.

**Table 3:** Multiple Dose Pharmacokinetics of GSK2838232 with and without Ritonavir—Study 204953

Dose (mg)	N	*t*½ (h)	*t*_max_ (h)	*C*_max_ (ng/mL)	AUC(0‐τ)^1^ (ng·h/mL)	Cτ^1^ (ng/mL)	CL/F (L/h)
Study day 1							
20/r (PiB)	6	ND	3.74 (3.00‐4.00)	11.1 (6.67, 18.5)	175 (109, 282)	6.09 (2.48‐8.94)	ND
50/r (PiB)	6	ND	2.50 (2.00‐6.00)	17.1 (10.2, 28.5)	265 (161, 438)	11.1 (5.11‐12.6)	ND
100/r (capsules)	6	ND	2.25 (1.50‐3.00)	46.2 (32.2, 66.2)	635 (448, 901)	22.7 (12.8‐29.3)	ND
200/r (capsules)	6	ND	2.25 (2.00‐3.50)	75.0 (51.4, 109)	1056 (704, 1586)	40.1 (21.7‐58.6)	ND
200 mg bid (capsules)	6	ND	3.74 (3.00‐6.00)	48.5 (28.6, 82.3)	267 (158, 452)	20.0 (9.23‐35.0)	ND
Study Day 11							
20/r (PiB)	6	19.8 (16.6, 23.7)	5.98 (2.50‐6.00)	25.3 (15.6, 41.1)	449 (297, 678)	17.0 (7.13‐19.2)	44.5 (29.5, 67.3)
50/r (PiB)	6	20.5 (14.7, 28.5)	3.75 (1.50‐6.00)	56.6 (44.0, 72.9)	1087 (844, 1401)	40.8 (24.5‐51.7)	46.0 (35.7, 59.2)
100/r (capsules)	6	17.7 (14.9, 21.1)	3.25 (2.50‐6.00)	130 (100, 168)	2411 (1782, 3263)	89.3 (45.9‐113)	41.5 (30.7, 56.1)
200/r (capsules)	6	18.0 (15.6, 20.8)	3.25 (1.50‐6.05)	225 (148, 341)	4014 (2442, 6599)	142 (67.8‐250)	49.8 (30.3, 81.9)
200 mg bid (capsules)	6	19.4 (14.7, 25.6)	3.76 (2.00‐6.00)	96.1 (66.0, 140)	742 (501, 1100)	38.4 (23.2‐61.8)	269 (182, 399)

Data presented as geometric mean (95% confidence interval) except *t*_max_ and Cτ, presented as median minimum‐maximum). ND, not determined. τ = 24 hours for QD dosing with RTV and 12 hours for BID dosing without RTV

RTV‐boosted GSK2838232 doses of 20‐200 mg QD achieved mean steady‐state C24 concentrations of 15‐150 ng/mL, which are 3‐ to 30‐fold of the protein‐adjusted IC90 (PA‐IC90) (5 ng/mL). The 200 mg BID unboosted dose achieved steady‐state C24 mean concentrations of 39 ng/mL (~8‐fold of the PA‐IC90).

### 3.4 Formulation studies and food effect

A comparison of the initial SDD and crystalline API PiB (Study 200912) indicated that the API had approximately 30%‐50% bioavailability relative to the SDD formulation. However, the API was considered more attractive for manufacturing and developability reasons, including a lower overall tablet weight compared to SDD. Therefore, the crystalline API, initially as PiB suspension and ultimately as capsules, was chosen for continuation of the clinical development program through Study 204953.

In Study 204953, total exposures of GSK2838232 following administration of a single 100 mg GSK2838232 dose (as two 50 mg capsules) in combination with RTV were approximately 1.4‐ to 1.5‐fold of those seen after higher than a corresponding 100 mg dose as API PiB suspension with RTV (Table 4). *C*_max_ and C24 values with the capsule formulation were 1.6‐fold and 1.4‐fold of those seen with the PiB suspension, and the geometric LS mean ratio (90% confidence interval) for t½ showed no difference between the two products (data not shown).

**Table 4:** Summary of results of comparisons for relative bioavailability of GSK2828232 API PIB versus API capsule formulations + Ritonavir—Study 204953)

Parameter	Comparison	N	Ratio of Geometric Least Square Means	90% CI of Ratio
AUC(0‐∞)	Capsule : API PiB	12	1.43	1.196, 1.702
*C*_max_	Capsule : API PiB	12	1.58	1.312, 1.900

When administered in the fasted state, GSK2838232 was rapidly absorbed, with time to *C*_max_ (*t*_max_) values of 2‐3 hours. Absorption was delayed and more variable after food. The bioavailability of the initial SDD PiB formulation after a high‐fat (60%) meal was approximately 1.6‐fold relative to the fasted state (Study HMI116787, Table 5), and the bioavailability of the capsule formulation given with RTV was approximately 1.6‐fold after a moderate fat (30%) meal relative to the fasted state (Study 204953, Table 5).

**Table 5:** Summary of food effect assessment on GSK2838232 PK parameters—studies HMI116787 and 204953

PK Parameter	Geometric least square mean ratio [90% CI]	
	50 mg Fed^a^ versus 50 mg fasted SDD PiB formulation (Study HMI116787)	100 mg Fed^b^ versus 100 mg fasted API capsule formulation (Study 204953)
AUC(0‐∞)	1.61 (1.266, 2.053)	1.58 (1.412,1.777)
*C*_max_	1.60 (0.995, 2.566)	1.62 (1.376,1.911)

a High (60% fat) meal

b Moderate (30% fat) meal

### 3.5 Safety

Nonclinical evaluation in short duration toxicology studies initially gave rise to potential cardiovascular signals which were minimal, appeared sporadically, and were not reproduced in longer term studies. Consequently, there was intensive and extensive cardiovascular monitoring at baseline and post‐dosing, including continuous Holter monitoring and regular cardiac troponin laboratory assessments in these early Phase I studies along with a high degree of vigilance for any findings and/or reported AEs of potential cardiovascular origin.

However, in these healthy volunteer studies, GSK2838232 demonstrated good tolerability when given as single doses up to 250 mg with 100 mg RTV and as repeat doses up to 200 mg with 100 mg RTV for 11 days, and no cardiovascular toxicities were deemed related to study drug administration.

Few adverse events (AEs) were reported across all dose regimens, and frequencies of AEs were similar to placebo. The majority of AEs were mild in intensity. Infrequently reported AEs assessed with a reasonable likelihood of being drug‐related were headache and dizziness. Sporadic cardiovascular events were seen on active and placebo arms, and none were considered definitively related to GSK2838232. There were no clinically significant changes in hematology or clinical chemistry laboratory values, including liver function tests, with the exception of one subject in Study 200912, who was subsequently diagnosed with gallstones (considered not treatment‐related). All other treatment‐emergent laboratory abnormalities were mild and not clinically significant.

Two cardiovascular findings were observed in Study 200207 in two different subjects: a mild troponin elevation in one subject, and a nonsustained ventricular tachycardia (NSVT) that was associated with dyspnea in another. One subject had a troponin elevation on the second day of dosing with 20 mg of GSK2838232. This event was not associated with symptoms, vital sign abnormalities, or ischemic changes on serial electrocardiograms. The Investigator did not consider the troponin elevation to be clinically significant. Normalization of troponin levels despite continued dosing suggested that this was likely not a drug‐related change. Another subject experienced a 14 beat/4 second run of NSVT 39 hours after the last of eight daily 50 mg doses of GSK2838232. The plasma concentration of GSK2838232 at that time of the event was <1 ng/mL. After a period of 17 weeks, the subject underwent continuous telemetry for 13 days with no drug (GSK2838232 or placebo) administration to assess underlying irregular cardiac electrophysiology. At a similar timeframe to that observed earlier, the subject experienced another run of NSVT, leading to the conclusion of a predisposition to this occurrence. The subject underwent an MRI of the heart 74 days after the last dose of study drug that revealed trace mitral regurgitation but otherwise normal heart findings. After extensive evaluations of these two cases and consultation with internal and external independent experts, it was concluded that, although the possibility of the relatedness of these events to GSK2838232 could not be completely excluded, the likelihood of a relationship was very low. However, these findings led to an FDA‐imposed clinical hold and the premature termination of Studies 200912 and 200207.

The follow‐up clinical data on these subjects together with completion of definitive chronic long‐term preclinical safety assessment studies (6‐month rat, 9‐month dog) led to the removal of the clinical hold and resumption of clinical dosing, as Study 204953. Intensive cardiovascular assessments with Holter monitoring and cardiac troponin laboratories were implemented in Study 204953 as well. Two subjects were withdrawn due to adverse events that were not considered related to GSK2838232: one subject was withdrawn due to two episodes of mild ventricular extrasystoles that occurred following RTV dosing but before GSK2838232 (100 mg) was administered in Period 3 (subject had received 50 mg + RTV in Period 2), and one subject was withdrawn due to as SAE of ventricular tachycardia while receiving placebo. Both events were of short duration and resolved within about a minute without intervention.

## 4 DISCUSSION

Administration of GSK2838232 to 124 healthy volunteers (115 males and 9 females not of child‐bearing potential) at single doses up to 250 mg, repeated doses of 200 mg twice‐daily, or repeated daily doses of up to 200 mg in combination with 100 mg RTV exhibited a good safety and tolerability profile, with few mild AEs reported across four clinical studies and no apparent trends in laboratory toxicities. There were no clearly identified drug‐related AEs. Concerns for elevated cardiovascular risk based on findings in the early preclinical studies were addressed in longer‐term preclinical studies and in these clinical studies.

In aggregate, the studies showed that a micronized API formulation of GSK2838232 in capsules was suitable for future clinical studies. All products were quickly absorbed with *t*_max_ of 2‐3 hours when administered in the fasted state. In the fed state (moderate or high fat meal), the absorption was delayed and more variable. The presence of food causes the release of bile that is expected to increase solubility of GSK2838232 due to bile surfactant properties. The transition to a capsule formulation included the addition of excipients (e.g., lactose and cellulose) as part of the blend (~75% of the total load), and the presence of these excipients is expected to delay GSK2838232 precipitation, allowing more drug to be in solution for absorption.^7^ This phenomenon is widely known as the “parachute effect”.^8^ Overall, the capsule formulation's enhanced bioavailability relative to the powder‐in‐bottle suspension and the positive food effect of about 1.5‐fold offered a significant improvement in overall exposure and, importantly, trough concentrations.

Generally, GSK2838232 AUC and *C*_max_ exhibited proportional pharmacokinetics over the dose range of 5 mg to 100 mg given as single doses without RTV and 50 mg to 250 mg when co‐administered as single doses with RTV. There was a less‐than‐proportional increase in exposure between 100 mg and 200 mg as single doses of the SDD formulation in Study 200912, suggesting solubility/dissolution and absorption limitation at higher doses with that formulation; the CL/F values are consistent with this interpretation. The relative bioavailability of the API was approximately 30%‐50% of the SDD formulation used in the first study, but, unlike the SDD formulation, the API displayed a proportional increase in exposure between the 100 mg and 200 mg dose levels in Study 200912.

RTV (following steady‐state predosing with 100 mg QD) increased exposure (AUC and *C*_max_) of GSK2838232 following a single dose by approximately 10‐fold and 3‐fold, respectively. The concept of pharmacokinetic boosting, where the metabolism of one drug is inhibited by another drug, is well‐documented with RTV and more recently cobicistat (both potent inhibitors of intestinal and hepatic CYP3A4 and P‐glycoprotein [P‐gp]) in order to improve the effectiveness and dosing convenience for anti‐HIV medications, especially HIV protease inhibitors.^9^, ^10^ Systemic exposure of GSK2838232 was significantly increased by co‐administration with RTV, with the only metabolite detected in human plasma an O‐glucuronide. The exact interplay and relative contribution to increased GSK2838232 bioavailability between hepatic and GI CYP3A4 inhibition and/or P‐gp inhibition by RTV co‐administration is unknown, but CYP3A4 and P‐gp have largely overlapping substrate specificities and are co‐localized in the intestine, working in a coordinated transport‐metabolism manner.^11^

Co‐administration of GSK2838232 and RTV together on Day 1 without ritonavir predosing showed only about a 25% reduction of Day 1 AUC and *C*_max_ value relative to the GSK2838232 AUC seen after the first GSK2939232 dose following predosing ritonavir to steady‐state. These data indicated that there is no need to predose with RTV in HIV patients, as GSK2838232 plasma concentrations attain potentially therapeutic levels within a few days.

Repeat dosing for 11 days with GSK2383232 QD at 20 and 50 mg as PiB or at 100 mg and 200 mg as capsules with RTV exhibited proportional pharmacokinetics for *C*_max_ and AUC. Steady‐state concentrations were achieved in 4‐7 days following once‐daily dosing of GSK2838232 with RTV. The observed values for *t*½ in Study 204953 after the last repeat dose of GSK2838232 with RTV (18‐22 h) were lower than those seen in earlier studies, in which RTV was administered for two further days during assessment of the GSK2838232 terminal phase (*t*½ values up to 30‐40 h), likely reflecting reduced CYP3A4 and P‐gp inhibition by RTV during the postdose sampling period.

Pharmacokinetic data in healthy subjects indicate that daily doses of 50‐200 mg in combination with RTV will achieve trough concentrations well above a minimum value of the PA‐IC90 (5 ng/mL). This initial target based on trough levels will require evaluation in clinical studies in HIV patients, which are ongoing. Overall, these early clinical development studies in healthy subjects have demonstrated GSK2838232 to have a good safety profile and to attain plasma concentrations that are likely to confer anti‐HIV clinical activity in future studies in HIV patients, either in the presence of RTV as once‐daily doses or potentially as higher twice‐daily unboosted doses.

## DISCLOSURE

This work and publication was sponsored and funded by GlaxoSmithKline. All authors are currently, or were employees of and owned stock in GSK at the time of study conduct.
